# Does Spousal Support Can Decrease Women’s Premenstrual Syndrome Symptoms?

**DOI:** 10.5539/gjhs.v8n5p19

**Published:** 2015-08-20

**Authors:** Hajar Rezaee, Fariba Mahamed, Maryam Amidi Mazaheri

**Affiliations:** 1 School of Health, Isfahan University of Medical Sciences, Isfahan, Iran; 2 Social Determinants of Health Research Center, Yasuj University of Medical Sciences, Yasuj, Iran

**Keywords:** intervention, premenstrual syndrome, social support perceived, spouse

## Abstract

Premenstrual syndrome is a syndrome that includes behavioral and physical symptoms occurring in the second half of the menstrual cycle and this syndrome affects millions of women universal. With regard to the importance of spouse participation in promoting reproductive and women’s health, the aim of this study was to determine the effect of educational intervention for spouse on women’s premenstrual syndrome symptoms. This quasi -experimental study was down with the participation of 100 women of reproductive age with PMS were referred to health centers Falavarjan city in 2015. Women were divided randomly into two groups as intervention and control. Educational intervention about supportive behaviors to control premenstrual symptoms was performed for spouses during the three educational sessions in the intervention group. Data was obtained with self-administered questionnaire before and three months after educational intervention and were analyzed by SPSS21 and appropriate statistical tests. Three mounts after the intervention the score of spouse’s supportive behaviors was increased significantly compare to before of the educational intervention and the control group. As well as significant decrease was occurred in case of physical and psychological-behavioral symptoms of women in the intervention group compare to before the intervention and control groups (p<0.05). Spouse’s supportive behaviors can reduce PMS symptoms in women. As a result, it is recommended that the health care system organize the educational intervention to increase spouse supportive behaviors.

## 1. Introduction

### 1.1 Introduce the Problem

Premenstrual syndrome is a syndrome that includes behavioral and physical symptoms occurring in the second half of the menstrual cycle and this syndrome affects millions of women universal, interfering in the quality of life, in the family and social relations, and in the performance of the daily living activities (Hoga, Vulcano, Miranda, & Manganiello, 2010). According to American College of Obstetricians and Gynecologists (ACOG), PMS is detected with present of at least one moderate to severe emotional or physical symptom (Balaha, Amr, Moghannum, & Muhaida, 2010). According to the ACOG, PMS symptoms include anxiety, anger, mood instability, feeling out of control, difficulty sleeping, change in appetite, fatigue, difficulty concentrating, chest pain, lethargy, muscle or joint pain, acne, weight gain, impaired adaptation and bloating (ali Morowatisharifabad, Karimiankakolaki, Bokaie, Fallahzadeh, & Gerayllo, 2014). In Balaha study the prevalence of PMS was 35.6%, distributed as 22.4% severe, 32.6% moderate and 45% mild (Balaha et al., 2010). In Samadi study the prevalence of this PMS in Iran was estimate as 62.4% and 67.7% (Samadi, Taghian, & Valiani, 2013).

### 1.2 Explore Importance of the Problem

Various studies revealed that more than 80% of females in reproductive age possibly will experience several physical and/or emotional premenstrual symptoms (Schiola, Lowin, Lindemann, Patel, & Endicott, 2011). Most women complain that emotional Changes due to PMS affect family and social relationships; specifically, 72% sense that PMS has a negative effect on the quality of the relationship with their spouses and other member of family (ali Morowatisharifabad et al., 2014). During life span prevalence of PMS may vary from 75 to 85% of women if an isolated symptom is measured, among 10 and 15% when women request medical support, and from 2% and 8% if disturbance of family and social activity is discussed (Pérez-López, Chedraui, Pérez-Roncero, López-Baena, & Cuadros-López, 2009). Another kind of PMS complications is indirect economic cost (Sorey, 2011). Other complications can include behavior changes, suicide attempts, criminal behavior, hospital admissions, absenteeism from work and increased prone to accidents can be aggravated premenstrual (Vishnupriya & Rajarajeswaram, 2011). Although the etiology of premenstrual symptoms is unknown and there is no certain cure for this syndrome, the treatment is symptomatic (Murakami et al., 2009). More than 300 various kind of medical, psychiatric, and cognitive treatment are suggested for premenstrual syndrome (Weisz & Knaapen, 2009). Studies have proven that with increase the level of social support the level of health increases and conversely (Riahi, Wardinia, & Pourhosein, 2011).

### 1.3 Describe Relevant Scholarship

Social support, as a coping emotion-oriented method can prevent the occurrence of stressful situations to protect people or help them to evaluate stressful events in a way that they look less threatening. Social support can be provided as psychological and emotional support and informative support but perceiving the provided support has special importance, in other words it seems that people’s understanding of received support is more important than the amount of the support which is provided (Mattila, Kaunonen, Aalto, Ollikainen, & Åstedt - Kurki, 2010; Reblin & Uchino, 2008).

Affective support may be known as the most important kind of support without considering the social network members who provide it. Affective support emphasizes empathetic relationships with social network members and includes verbal and care relations such as listening, empathizing and providing people with comfort resulting in increasing knowledge and awareness and coping skills, rebuilding self-confidence and decreasing incompetence feeling and negative feelings and as a result increasing sense of control in people. This kind of support allows people to express their feelings so their tensions decrease causing improved relationship (Heidari, 2009). This kind of support plays an important role in people’s health, so those who have social support have a better sense of self-efficacy and are more successful in most fields and behave more appropriately towards emotional interactions (Travaglia, Westbrook, & Braithwaite, 2009).

Support from important people like spouse, as a coping emotion-oriented method, can protect people bypreventing the occurrence of stressful situations or can help them evaluate stressful events such as PMS in a way that they seem less threatening (Reblin & Uchino, 2008). Generally, men did not have enough knowledge on the effects of the PMS in women. Even when men want to provide support to women being affected by the syndrome they get lost because they do not know what to do. The spousal relationship can be tremendously affected by this lack of suitable knowledge; consequently the sexuality, the marriage situation, and women’s satisfaction with their relatives were better when their spouses were alert of the effects of the PMS on their partners’ minds and bodies (Hoga et al., 2010).

Previous study revealed that PMS affects in the life of couples, particularly in their communication. In the premenstrual period, the discourse among spouses diminished, and the strength of it was associated with the severity of symptoms and signs existing by females (Hoga et al., 2010).

### 1.4 State Hypotheses and Their Correspondence to Research Design

Hence men to do supportive behavior should be aware of the changes suffered by women and recognize women’s problems, so their attitudes can change and the quality of relationship between them can modification. Educating men about PMS is still a challenge that reproductive health professionals should embrace. Hence health and education specialists should create suitable opportunities for men to take part in discussions about issue (Hoga et al., 2010).

Consequently, increasing spouses’ knowledge in case of reproductive health especially premenstration syndrome is important and additional studies in this are needed (Dean, Borenstein, Knight, & Yonkers, 2006).

The psychological problems of premenopausal syndrome not only involve women but also, in the majority of cases, backfire on their spouses through their complications. In fact, men’s mistreatment towards the changes of mood in wives is one of the greatest psychological worries of women. Spouse’s unawareness and inappropriate behavior, while his wife is undergoing premenopausal syndrome, lead to conflicts in the family. Subsequently considering lack of researches on this field in Iran and the high prevalence of premenopausal syndrome in married women during reproductive ages and insufficient men’s knowledge of this issue, the present study has reviewed this question: “Does spousal’ support can decrease women’s premenstrual syndrome symptoms.”

After you have introduced the problem and have developed the background material, explain your approach to solving the problem. In empirical studies, this usually involves stating your hypotheses or specific question and describing how these were derived from theory or are logically connected to previous data and argumentation. Clearly develop the rationale for each. Also, if you have some hypotheses or questions that are central to your purpose and others that are secondary or exploratory, state this prioritization. Explain how the research design permits the inferences needed to examine the hypothesis or provide estimates in answer to the question.

## 2. Method

### 2.1 Identify the Study

The present research is a semi-experimental study conducted on 20-45 year old married women in Falavarjan city. Simple random sampling was used.

### 2.2 Participant Characteristics

Inclusion criteria included: 20-45 year old women with premenopausal syndrome, living with spouse, normal menstrual cycle occurring every 21 to 35 days and lasting 3 to 10 days, spouse’s interest in participating in educational intervention; and exclusion criteria included women with physical and psychological diseases, taking medicines and complementary medicines or hormonal compounds, smoking and drug abuse, extreme stress like death of close relatives or having surgery during the past three months, and spouse’s one-session absence from the educational intervention.

### 2.3 Sampling Procedures

At first, after receiving a letter from the Faculty Vice-chancellor for Research and coordinating with Falavarjan’s Health care system, 190 families were selected as selective samples through family numbers in 12 available centers in Falavarjan. After introducing herself and giving required information about the study, the researchers gave the questionnaires and consent in packets to the people and ask them to complete the questionnaires and deliver them to their healthcare center. Out of eligible people, 100 were selected and divided randomly into two groups.

### 2.4 Measurments

Information related to physical and mental-behavioral symptoms was collected by premenopausal syndrome assessment questionnaire called PSA (Premenstrual syndrome assessment). This questionnaire, as a valid questionnaire, has been established by Delara and colleagues based on Iran society culture and its validity and reliability have been confirmed (Delara et al., 2013). This questionnaire includes two sections: the first section measures personal characteristics and the second one has 32 items and measures physical symptoms (14 items) and mental-behavioral symptoms (18 items) related to premenopausal syndrome. Each item was given a score ranging between 0 to3 (0: asymptomatic, 1: mild, 2: moderate, 3: severe). American College of Obstetricians and Gynecologists (ACOG) criteria was used to diagnose premenopausal syndrome. spouse s’ supportive behaviors were investigated by self-efficacy questionnaire, and the panel of experts method was used to assess the validity, that is, the first copy was given to 7 professors of health education and midwifery and its primary validity was investigated and they were asked for comments on the questionnaire and their comments were applied to the questionnaire (the significance level was regarded 0.05). Then this questionnaire was given to 20 women and its reliability which was obtained by Cranach’s alpha was 0.86. This questionnaire had 14 items out of which 7 items were related to the period before menstruation and 7 items were related to menstrual period. The items were scored in the range of 0 to 3 (0: no, not at all; 1: yes, sometimes; 2: yes, often; 3: yes, always).

### 2.5 Educational Interventions

To educate the spouses in the intervention group, the participants were arranged into 15-person groups and the educating course was done using educational films, group discussions, and question and answer method for three 1-hour sessions in Health and Care Center Hall on holidays.

In the first session, using educational films, the talk was about premenopausal syndrome symptoms; in the second session it was about general recommendations to spouses for controlling PMS in their wives; and in the third session it was about spouse s’ supportive roles in controlling these symptoms; and in the last session a general summary of the educational subjects which were mentioned in the previous sessions was provided. Three months after holding the meetings, the questionnaires were given to the women in the intervention and control groups and they were asked to complete them and give them to their healthcare centers. During this time, some short messages were sent to the spouses by the researcher to remind them of the educational subjects.

### 2.6 Statistics and Data Analysis

The data was analyses by the statistical software SPSS21; and t-test, t-pair test, Mann-Whitney, and Chi- square were used.

## 3. Results

the mean and standard deviation of women’s age in the intervention and control groups were 28.76±5.51and 30.38±5.31 respectively in the range of 20-45, and the mean and standard deviation of men’s age in the intervention and control groups were 33.36±5.66 and 35.58±5.84 respectively in the range of 23-57 and no statistically significant difference was observed between the mean ages of women and men in two groups P<0.001. Other characteristics of the participants in both groups are detailed in Table 1. The two groups had no significant differences in demographic variables.

**Table 1:** Comparing the demographic variable of participants in the intervention and control group

		Intervention group		control group		
	
		N	%	N	%
Educational status of women						
	Lower than school diploma	10	20	12	24	P<0.001
	Diploma and higher	40	80	38	76	
Educational status of men						
	Lower than school diploma	17	34	23	46	P<0.001
	Diploma and higher	33	66	27	54	
Women’s job						
	Housewives	40	80	43	86	P<0.001
	Employees	10	20	7	14	
Men’s job						
	Worker	14	28	24	48	P<0.001
	Self-employed	29	58	22	44	
	Office-worker	7	14	3	6	
	jobless	0	0	1	2	
Income						
	Lower than 800000 tomans	15	30	23	46	P<0.001
	800000 to 1000000 tomans	25	50	22	44	
	Higher than 1000000 tomans	10	20	5	10	

Considering the inclusion criteria to take part in the study based on ACOG criteria, the general prevalence of premenopausal syndrome before educational intervention was 100% in both groups which was estimated at 86% in the intervention group and 95% in the control group three months after educational intervention.

The information about PMS is presented in Table 2. Three months after the intervention the scores of physical and mental-behavioral symptoms of PMS in the intervention group were significantly recorded less than in the control group. After the educational intervention the scores of spouse support both during premenstrual and menstrual period were increased significantly in intervention group. Details of changes in spouse support are presented in Table 3.

**Table 2:** Comparing the scores of physical and mental-behavioral PMS symptoms in the intervention and control group before and three months after educational intervention

PMS* symptoms		intervention group N=50 SD±M	control group N=50 SD±M	t-test
Physical symptoms	Before intervention	6.57±8.62	4.50 ±6.44	P=0.056

	three months after intervention	3.65 ±3.82	5.08 ± 6.04	P=0.014

t-pair test		*P*<0.001	P=0.52	

mental-behavioral symptoms	Before intervention	7.48±8.44	6.45±7.20	P=0.37

	three months after intervention	3.54±3.24	5.67±6.60	*P*<0.001

t-pair test		*P*<0.001	P=0.49	

PMS	Before intervention	12.76±17.06	9.83±13.64	P=0.137

	three months after intervention	6.23±7.06	9.25 ±12.64	*P*<0.001

t-pair test		*P*<0.001	P=0.42	

* Premenstrual Syndrome.

**Table 3:** Comparing the mean scores of perceived spouse’s support in the intervention and control group before and three months after educational intervention

Scores of perceived spouse’s support about premenopausal syndrome		intervention group 50=N SD± M	control group 50=N SD± M	t-test
Support a week before menstruation	Before intervention	4.21±8.78	4.23±7.34	P=0.091

	Three months after intervention	4.31±13.66	4.49±7.64	*P*<0.001

t-pair test		*P*<0.001	P=0.53	

Support during menstruation	Before intervention	4.10±9.18	4.45±7.76	P=0.10

	Three months after intervention	3.99±14.18	4.09±7.82	*P*<0.001

t-pair test		*P*<0.001	P=0.90	

Total support	Before intervention	8.09±17.96	8.40±15.10	P=0.086

	Three months after intervention	8.16±27.84	8.31±15.46	*P*<0.001

t-pair test		*P*<0.001	P=0.69	

## 4. Discussion and Conclusion

Reproductive health has appeared as a structural frame work that includes male’s participation into maternal and child health (MCH) programmers and rising investigation. At the Global Discussion on Populace and Growth in Cairo 1994 and at the Quarter Conference of Females in Beijing 1995, men’s generative tasks have received total consideration. Resolves of together conferences encouraged for men’s communal duty, raise of their dynamic participation in accountable parenting, erotic and generative health performance (Secka, 2010).

To reach spouse participation, suitable efforts must be made by the whole society, especially by health, communication and education professionals.

The present study has investigated this question: Does spousal support can decrease women’s PMS symptoms?

The results of present study revealed that the scores of physical and mental-behavioral symptoms of PMS and perceived spouse’s support in both groups did not have statistically significant differences before educational intervention and all the women who were studied had PMS according to ACOG criteria but three months after the intervention the scores of physical and mental-behavioral symptoms of PMS in the intervention group were significantly recorded less than in the control group. After the intervention according to ACOG criteria the prevalence of PMS was decreased significantly in the intervention group. Based on some limited studies conducted on social support related to PMS, our finding was similar to Heywood and Hoga’s finding (Haywood, Slade, & King, 2007).

Our finding also was contrary with some studies for instance. Gulact study revealed that perceived social support from special people and friends does not affect participants’ well-being (Gülaçtı, 2010).

This discrepancy emphasizes the important of spouse support instead of special people and friends.

Several studies have revealed that one of the suitable ways to transfer educational subjects to women is their spouses which is one of the points that World Health Organization has emphasized to promote men’s participation in maintenance and promoting women’s health during reproductive ages, for example Sharif Rad and colleagues (2011) indicated in their study that training men in order to increase their wives’ knowledge and modify their attitudes toward type of delivery is effective and this training has been effective to decrease the amount of cesarean without medical reasons (Sharifirad, Rezaeian, Soltani, Javaheri, & Mazaheri, 2013).

Evidence suggests that most investigations have been done into women during reproductive ages rather into their spouses, for example studies on women during reproductive ages reveal that after educational intervention the scores of PMS symptoms in intervention groups have been lower than in control groups (Ramya, Rupavani, & Bupathy, 2014; Saxena, Kumari, Khurana, & Rawat, 2014). Also previous studies have shown that women who use social support experience less severe PMS (Ussher & Perz, 2013), and that discussion about premenstrual experiences and requirements can improve distress (Mooney-Somers, Perz, & Ussher, 2008). Emotional support from friends and family member has been related through reports of lesser PMS symptoms and more effective coping and better tolerance (Ussher & Perz, 2013).

The present study results showed that educating spouses in the intervention group led to an increase in supportive behaviors scores and decrease in physical and mental-behavioral symptoms of PMS of their women and this change in mental-behavioral symptoms was stronger, so based on the resultsof this study it can be concluded that training spouses has been useful to decrease PMS symptoms.

Social support provided by the members of family has a main role in reducing stress factors and solving the psycho-social problems, in controlling the problems before arising, in protecting family member’s psychological health and therefore confirming the extension of family harmony (Mehmet, 2012).

Family members especially spouses are considered as the most important lifelong sources of relational support for the individual. While only marriage relations are considered, spouses are commonly more important than other sources of social support as providers of support for women (Mehmet, 2012).

In fact, the spouse is the most important and closest person who can support his wife by appropriate understanding of the situation and help her to control the physical and mental-behavioral symptoms of premenopausal syndrome. Women need to be perceived and understood to protect their mental and affective health. Spouse s’ attention to this basic need by listening to their wives’ words and understanding their feelings causes improvement in women’s affective and psychological health.

In fact, one of the needs that are present in women’s lives is intimacy, dependency, sense of being understood and approved. These needs appear in the form of desire to build up friendly relationships with matches especially with mother during childhood and adolescence and in the form of friendship along with mutual understanding of affective relationships during youth. Men can provide suitable conditions to support their wives affectively through encouraging them. Men can listen to their wives’ talks, chats, and issues and allow them to express their feelings. Most of the men who are familiar with premenopausal syndrome know that affective support for their wives can solve a lot of problems and in fact they understand that this phenomenon is out of control and it is possible to prevent undesirable events by a little help and companionship.

Men’s participation promotion as a principle, which is emphasized by world health organizations, is possible by training in order to promote women’s health. According to the findings of this study it is suggested that suitable educational programs should be planned to promote men’s supportive behaviors during a week before menstruation and during menstruation.

The present study was one of the few studies which investigated spouse s’ supportive behaviors and their effects on PMS and had some limitations such as the small number of samples, retrospective questionnaire completion and telephone completion of the questionnaires which were not delivered to healthcare centers. So for more decisive stance on the relationship between perceived social support and PMS status, it is offered to conduct more studies with more samples through prospective methods.

As previous studies revealed (Mehmet, 2012), after educational intervention for men, the support spouses from women increased and conflicts between the couples reduced, and promoting spousal support could lead to decrease physical and mental-behavioral symptoms of premenopausal syndrome among women.
